# Mid‐ventricular obstruction is associated with non‐sustained ventricular tachycardia in patients with hypertrophic obstructive cardiomyopathy

**DOI:** 10.1002/clc.23575

**Published:** 2021-02-24

**Authors:** Changrong Nie, Changsheng Zhu, Minghu Xiao, Qiulan Yang, Yanhai Meng, Rong Wu, Shuiyun Wang

**Affiliations:** ^1^ Department of Cardiovascular Surgery Fuwai Hospital, National Center for Cardiovascular Diseases, Chinese Academy of Medical Sciences and Peking Union Medical College Beijing China; ^2^ Department of Ultrasound Fuwai Hospital, National Center for Cardiovascular Disease, Chinese Academy of Medical Sciences and Peking Union Medical College Beijing China; ^3^ Department of Intensive Care Unit Fuwai Hospital, National Center for Cardiovascular Diseases, Chinese Academy of Medical Sciences and Peking Union Medical College Beijing China

**Keywords:** hypertrophic cardiomyopathy, hypertrophic obstructive cardiomyopathy, mid‐ventricular obstruction, non‐sustained ventricular tachycardia

## Abstract

**Background:**

Mid‐ventricular obstruction (MVO) is a rare subtype of hypertrophic cardiomyopathy (HCM) but it is associated with ventricular arrhythmia. The relationship between MVO and non‐sustained ventricular tachycardia (NSVT) in HCM patients is unknown.

**Hypothesis:**

The severity of MVO increases the incidence of NSVT in patients with hypertrophic obstructive cardiomyopathy (HOCM).

**Methods:**

Five hundred and seventy‐two consecutive patients diagnosed with HOCM in Fuwai Hospital between January 2015 and December 2017 were enrolled in this study. Holter electrocardiographic and clinical parameters were compared between HOCM patients with and without MVO.

**Results:**

Seventy‐six (13.3%) of 572 patients were diagnosed with MVO. Compared to patients without MVO, those with MVO were much younger, and had a higher incidence of syncope, greater left ventricular (LV) posterior wall thickness, a higher percentage of LV late gadolinium enhancement, and higher prevalence of NSVT. Furthermore, the prevalence of NSVT increased with the severity of MVO (without, mild, moderate or severe: 11.1%, 18.2%, 25.6%, respectively, p for trend < .01). Similarly, the prevalence of NSVT differed among patients with isolated LV outflow tract (LVOTO), both MVO and LVOTO, and isolated MVO (11.1%, 21.3%, 26.6%, respectively, p for trend = .018). In addition to age, diabetes, left atrial diameter, and maximal wall thickness, multivariate analysis revealed the presence of MVO as an independent risk factor for NSVT (Odds ratio 2.69; 95% confidence interval 1.41 to 5.13, p = .003).

**Conclusions:**

The presence and severity of MVO was associated with higher incidence of NSVT in HOCM patients.

## 1 INTRODUCTION

Hypertrophic cardiomyopathy (HCM) is a common inherited cardiac disease with a prevalence of approximately 0.2%–0.5% in the general population.^1^ The majority of HCM patients have a left ventricular outflow tract obstruction (LVOTO) at rest or with provocation.^2^ HCM patients with mid‐ventricular obstruction (MVO), impedance to flow at the middle of the left ventricle, is a less common subtype of HCM but it is associated with ventricular arrhythmia and a worse prognosis.^3^, ^4^, ^5^ However, most studies concerning the relationship between HCM with MVO and ventricular arrhythmia were case reports or small series.^6^, ^7^, ^8^, ^9^, ^10^, ^11^Moreover, no studies have focused on the relationship between HCM with MVO and non‐sustained ventricular tachycardia (NSVT), which was a strong predictor of sudden cardiac death in patients with HCM. The hypothesis of this study was that MVO severity affects NSVT incisence in patients with hypertrophic obstructive cardiomyopathy (HOCM).

## 2 MATERIALS AND METHODS

### 2.1 Population

We retrospectively reviewed 857 consecutive patients who diagnosed with HOCM at Fuwai Hospital (Beijing, China) between January 2015 and December 2017. Of the 857 patients, 572 who underwent 24 h Holter electrocardiography were finally enrolled in this study. All patients were divided into two groups: patients with MVO (N = 76) and those without MVO (patients with isolated LVOTO) (N = 496), according to the echocardiography results. Holter electrocardiographic and clinical parameters were compared between HOCM patients with and without MVO. Furthermore, to illustrate the relationship between the mid‐ventricular gradient and the prevalence rate of NSVT, we divided patients with MVO into two subgroups based on the mid‐ventricular gradient: mild, 30–50 mmHg (N = 33); and moderate or severe, >50 mmHg (N = 43). In addition, the prevalence rate among patients with isolated LVOTO, both LVOTO and MVO, and isolated MVO was also analyzed.

**FIGURE 1:**
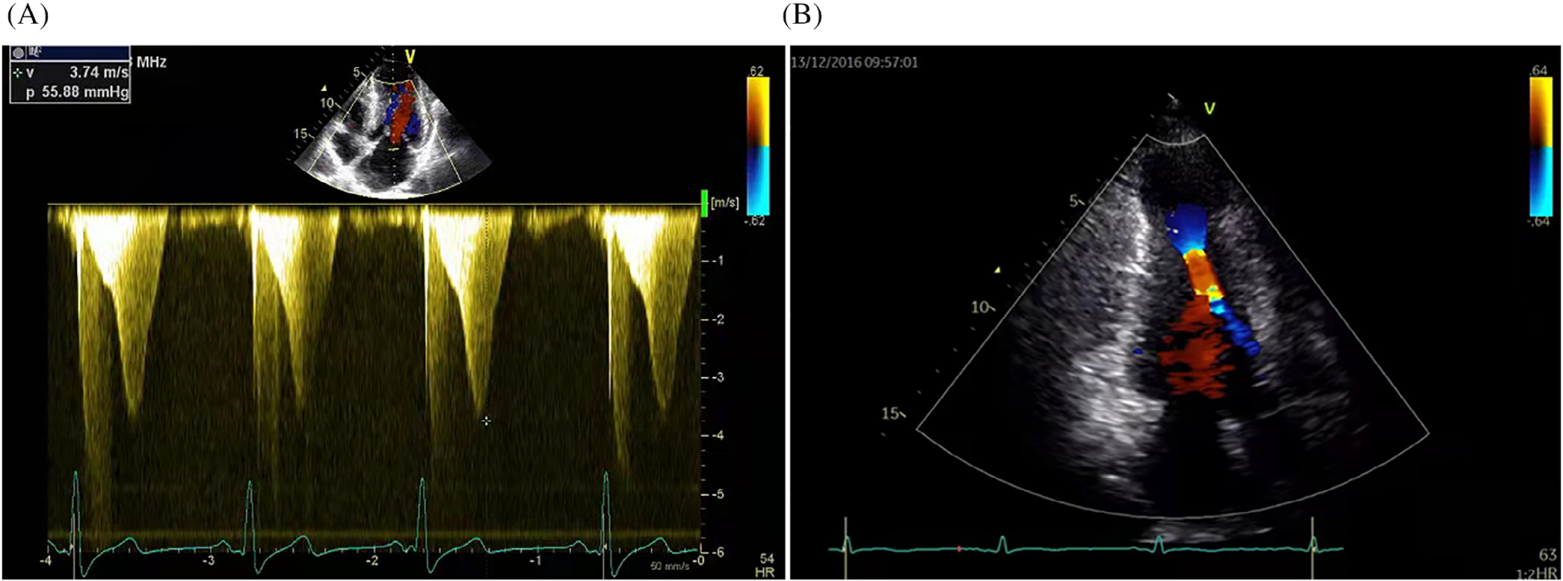
(A) A dagger‐shaped waveform is obtained, showing a high mid‐ventricular gradient, and the estimated peak velocity of 3.74 m/s. (B) Two‐dimensional transthoracic echocardiography from the LV long‐axis two‐chamber view showing an hourglass shape of the left ventricle during systole. LV, left ventricular

The diagnosis of HOCM was based on the 2020 American Heart Association/American College of Cardiology guideline and the 2014 European Society of Cardiology guideline, which mainly included unexplained septal hypertrophy with a thickness > 15 mm or a thickness of septal cardium >13 mm with a family history of HCM.^12^, ^13^ The indication for LVOT obstruction was an LVOT gradient ≥30 mmHg at rest or with provocation. MVO was defined as peak mid‐ventricular gradient ≥30 mmHg, with a simultaneous appearance of mid‐ventricular muscular apposition, causing an hourglass shape of the left ventricle on echocardiography (Figure 1).^4^, ^14^

All study participants signed informed consent forms before enrollment, and this study was approved by the Ethics Committee of Fuwai Hospital. All procedures of this study were conducted in accordance with the ethical principles stated in the Declaration of Helsinki.

### 2.2 Twenty‐four‐hour Holter electrocardiography

Twenty‐four‐hour Holter electrocardiogram was performed for all study subjects. These data were technically of good quality, and rhythm and arrhythmia were analyzed conventionally. NSVT was defined as an episode of ≥3 consecutive ventricular beats with a rate of at least 100 beats/min, lasting for <30 s. Each NSVT episode was recorded for frequency, duration, and maximal rate. Couplets were two consecutive premature ventricular complexes (PVCs). More details can be found in our previous publication.^15^

### 2.3 Echocardiography

Transthoracic echocardiographic studies were performed using a commercially available system (E9 ultrasound system, GE Healthcare, Horten, Norway). With patients in the left lateral decubitus or supine position, complete M‐mode echocardiography, two‐dimension echocardiography, and Doppler studies were performed using standard parasternal, apical, and subcostal approaches. The diameters of the cardiac chambers were presented as the maximum value of the anteroposterior diameter in cardiac cycles. Maximum left ventricular (LV) wall thickness was the greatest dimension measured at any site within the LV chamber at end diastole. The LVOT gradient was scanned with continuous‐wave Doppler to measure maximal outflow velocity and estimated using the simplified Bernoulli equation. MVO refers to apposition of the mid‐ventricular walls during systole and often the papillary muscles with abnormally high‐velocities persisting through late systole and usually with early diastolic paradoxical jet flow.^4^

### 2.4 Statistical analysis

The results are presented as mean ± standard deviation or number (percentage), as appropriate. Student's *t*‐test for independent samples and the χ2 or Fisher's exact test were used to compare nominal variables, as appropriate. Univariate and stepwise multivariate logistic regression analyses were used to determine factors associated with NSVT. Variables with a p < .1 in univariate analysis or previously demonstrated to be associated with NSVT were entered into a multivariate analysis. All p values were two‐tailed, with a p value < .05 indicating statistical significance. SPSS version 26.0 (IBM) and GraphPad Prism version 8.0 (GraphPad Software Inc., La Jolla, CA) were used for calculation and illustration, respectively.

## 3 RESULTS

### 3.1 Baseline patient characteristics

In the present study, 76 (13.3%) patients were diagnosed with MVO, and 61 of 76 patients had both MVO and LVOTO. The mean age of patients with MVO at diagnosis was 44.6 ± 12.2 years, and the mean mid‐ventricular gradient was 52.7 ± 17.1 mmHg. The clinical characteristics of patients with and without MVO are summarized in Table 1. Patients with MVO were significantly younger and had a lower LVOT gradient, higher prevalence rate of aneurysms, and greater LV posterior wall thickness than those without MVO. The history of syncope in patients with MVO was significantly higher than in patients without MVO. Furthermore, 296 patients underwent cardiac magnetic resonance, the percentage of LV late gadolinium enhancement (LGE) was also significantly higher in patients with MVO than those in without MVO.

**TABLE 1:** Baseline patient characteristics

Variables	HOCM with MVO (N = 76)	HOCM without MVO (N = 496)	p
Male (N, %)	37 (62.7)	297(57.9)	.742
Age (year)	44.6 ± 12.2	49.4 ± 12.3	.002
Heart rate (beats/min)	71.1 ± 10.0	71.5 ± 9.2	.701
Systolic blood pressure (mmHg)	124.3 ± 17.5	122.4 ± 14.8	.323
Diastolic blood pressure (mmHg)	74.4 ± 11.4	72.9 ± 10.3	.231
BMI (kg/m2)	25.0 ± 3.5	25.7 ± 3.6	.118
Smoking (N, %)	29 (38.2)	196 (39.5)	.821
FH of HCM or SCD (N, %)	10 (13.2)	67 (13.7)	.903
Previous heart Surgery (N, %)	4 (6.8)	27 (5.3)	.626
NYHA class	2.78 ± 0.45	2.83 ± 0.47	.324
Clinic presentation			
Chest pain (N, %)	30 (50.8)	201 (39.2)	.084
Amaurosis (N, %)	17 (22.4)	104 (21.0)	.781
Syncope (N, %)	27 (35.5)	120 (24.2)	.035
Palpitation (N, %)	20 (26.3)	147 (29.6)	.553
Concomitant disease			
Diabetes (N, %)	4 (5.3)	25 (5.0)	.934
Hypertension (N, %)	23 (30.3)	144 (29.0)	.826
Hyperlipemia (N, %)	22 (28.9)	153 (30.8)	.738
Atrial fibrillation (N, %)	11 (14.5)	64 (13.0)	.720
Aneurysm	4 (5.3)	3 (0.6)	.001
LV LGE percent (%)	13.3 ± 8.3 (N = 46)	10.1 ± 9.2 (N = 250)	.031
Echocardiographic indices			
Left atrial diameter (mm)	45.1 ± 6.8	45.4 ± 7.1	.703
LVEDD (mm)	41.4 ± 4.6	42.6 ± 5.6	.063
IVST (mm)	18.9 ± 3.6	19.8 ± 9.1	.399
LVPWT (mm)	12.6 ± 3.3	11.8 ± 2.5	.013
MLVWT (mm)	22.6 ± 4.2	22.0 ± 4.8	.343
LVOT gradient (mm Hg)	59.5 ± 30.8	86.0 ± 26.0	< .001
LVEF (%)	70.8 ± 5.1	69.8 ± 6.0	.174
Moderate or severe MR (N, %)	25 (32.9)	187 (37.7)	.419

*Note*: Values are presented as mean ± SD or as N (%).

Abbreviations: BMI, body mass index; FH, family history; HCM, hypertrophic cardiomyopathy; HOCM, hypertrophic obstructive cardiomyopathy; IVST, interventricular septal thickness; LVEDD, left ventricular end‐diastolic diameter; LVEF, left ventricular ejection fraction; LVOT, left ventricular outflow tract; LVPWT, left ventricular posterior wall thickness; MLVWT, maximal left ventricular wall thickness; MR, mitral regurgitation; MVO, mid‐ventricular obstruction; NYHA, New York Heart Association; PAH, pulmonary artery hypertension; SCD, sudden cardiac death.

### 3.2 Holter electrocardiographic data

**FIGURE 2:**
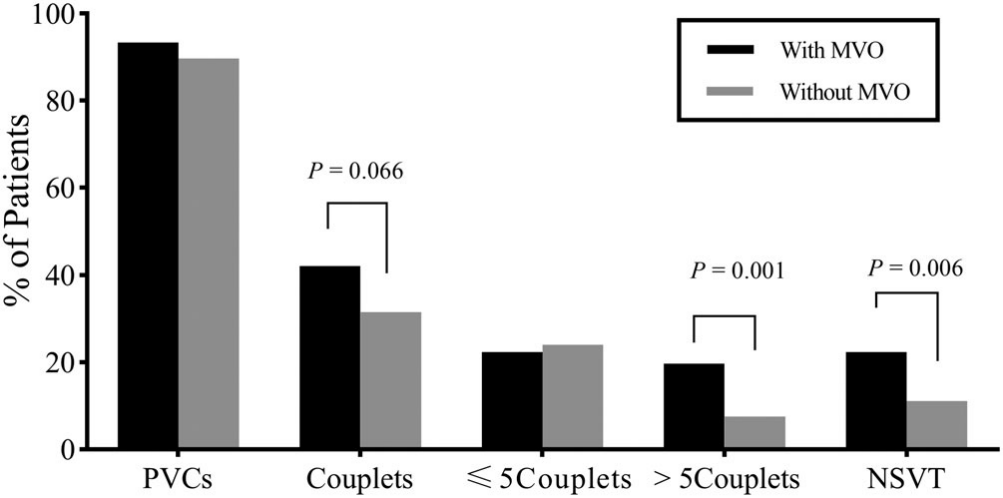
Prevalence of ventricular arrhythmias on 24‐h Holter electrocardiogram recording in patients with and without MVO. MVO, mid‐ventricular obstruction; NSVT, non‐sustained ventricular tachycardia; PVCs, premature of ventricular complexes

**FIGURE 3:**
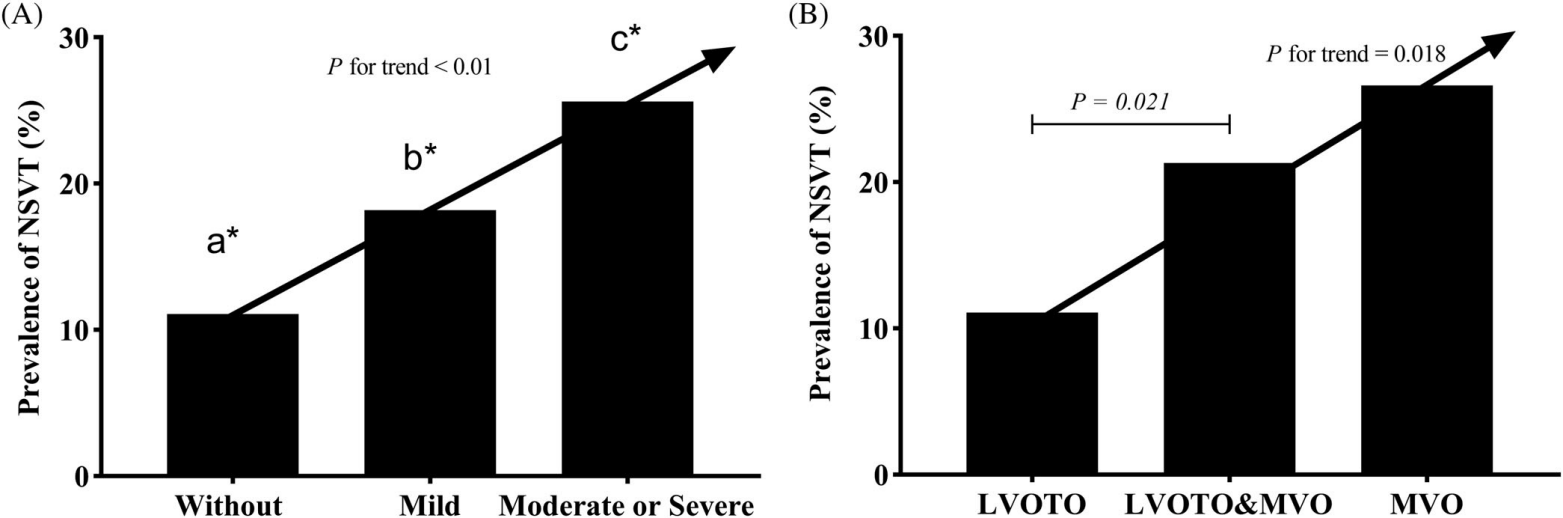
(A) The relation between the severity of MVO and occurrence of NSVT on 24‐h Holter electrocardiogram (p < .01 by the chi‐square test for trend). a*, b*, and c* means the difference between the three groups are all significant, all p‐value < .05. (B) The relation between patients with isolated LVOTO, both MVO and LVOTO, and isolated MVO and prevalence of NSVT on 24‐h electrocardiogram (p = .018 by the chi‐square test for trend). LVOTO, left ventricular outflow tract obstruction; MVO, mid‐ventricular obstruction; NSVT, non‐sustained ventricular tachycardia

Ventricular arrhythmia was common in HOCM patients and > 90% of patients had PVCs in this study. Patients MVO had a higher prevalence of >5 couplets of PVCs (19.7% vs. 7.5%, p = .001) and NSVT than those without MVO (22.4% vs. 11.1%, p = .006) (Figure 2).When patients with MVO were divided into mild and moderate or severe groups, patients with moderate or severe MVO had a significantly higher prevalence rate of NSVT than those with mild MVO (25.6% vs 18.2, p < .05) and the prevalence rate of NSVT increased with the severity of MVO (without, mild, moderate or severe: 11.1%, 18.2%, 25.6%, respectively, p for trend <.01) (Figure 3(A)). Similarly, the prevalence rate of NSVT also differed among patients with isolated LVOTO, both MVO and LVOTO, and isolated MVO (11.1%, 21.3%, 26.6%, respectively, p for trend = .018.) (Figure 3(B)).

### 3.3 Clinical data associated with NSVT

The variables with a p < .1 in univariate analysis or previously demonstrated to be associated with NSVT are presented in Table 2. In the multivariate analysis, in addition to age (odds ratio [OR] 1.03, 95% confidence interval [CI] 1.004–1.05, p = .020), diabetes (OR 2.59, 95% CI 1.03–6.12, p = .043), left atrial diameter (OR 1.04, 95% CI 1.01–1.08, p = .019), and maximal LV wall thickness (OR 1.13, 95% CI 1.06–1.19, p < .001), the presence of MVO (OR 2.69, 95% CI 1.41–5.13, p = .003) was found to be an independent risk factor for NSVT.

**TABLE 2:** Logistic regression models for analysis of the risk factors of NSVT

Variables	Univariate (OR, 95% CI)	p	Multivariate (OR, 95% CI)	p
Age	1.02 (0.99–1.04)	.165	1.03 (1.004–1.05)	.020
AF	1.87 (0.94–3.71)	.074		
Diabetes	2.85 (1.21–6.71)	.016	2.59 (1.03–6.12)	.043
FH of HCM or SCD	1.32 (0.67–2.59)	.418		
LAD	1.05 (1.01–1.08)	.011	1.04 (1.01–1.08)	.019
MVO	2.31 (1.26–4.24)	.007	2.69 (1.41–5.13)	.003
MLVWT	1.10 (1.04–1.15)	< .001	1.13 (1.06–1.19)	< .001

Abbreviations: CI, confidence interval; FH, family history; HCM, hypertrophic cardiomyopathy; MLVWT, maximal left ventricular wall thickness; MVO, mid‐ventricular obstruction; NSVT, non‐sustained ventricular tachycardia; OR, odds ratio; SCD, sudden cardiac death.

### 3.4 NSVT characteristics in patients with and without MVO

Seventy‐four patients (12.9%) had NSVT during their initial Holter electrocardiogram recordings. The mean number of runs of NSVT during the 24‐h Holter electrocardiogram was 4.4 ± 11.7 (range, 1–97). The mean number of beats in the longest run was 7.0 ± 6.0 beats (range, 3–35 beats) and the mean maximum rate of the NSVT runs was 132.0 ± 23.1 beats/min. NSVT characteristics of patients with and without MVO are presented in Supplemental Table 1.The mean runs, duration, and maximal heart rate of NSVT episodes did not differ between patients with and without MVO.

## 4 DISCUSSION

The main findings of this study are as follows: first, the prevalence rate of MVO in this study was approximately 13.3%. Second, the presence and severity of MVO significantly associated with a higher incidence of NSVT. Third, in addition to age, diabetes, left atrial diameter, and maximal wall thickness, the presence of MVO was found to be an independent risk factor for NSVT.

Overall, 13.3% of HOCM patients in the present single‐center cohort study had MVO. The frequency of MVO was similar to that noted in previous studies, which had reported the incidence of MVO to be between 8% and 12.9% among HCM patients.^3^, ^4^, ^16^, ^17^Due to the low‐prevalence rate of MVO in HCM patients, there is a paucity of information on the link between NSVT and MVO. Some case reports have shown that mid‐ventricular obstructive HCM with apical aneurysm usually presented with ventricular arrhythmia.^6^, ^7^Moreover, some small series indicated that there was a potential relationship between MVO and ventricular arrhythmia in HCM patients.^9^, ^18^ These studies showed a high prevalence rate of NSVT in HCM patients with MVO. Clinical studies of HCM with MVO are limited. Cai and colleagues analyzed 60 HCM patients with MVO, and their results demonstrated that patients with MVO had a higher incidence of NSVT and were more likely to have myocardial fibrosis on cardiac magnetic resonance imaging than patients with apical HCM.^19^ In addition, two other large single‐center studies showed that patients with MVO had a higher incidence of sudden cardiac death and potential lethal arrhythmia events than those with isolated LVOT obstruction.^3^, ^4^ These studies suggested that patients with MVO were potentially associated with a higher incidence of NSVT than those without MVO (patients with apical HCM or HCM patients with isolated LVOTO). In our present study, the Holter electrocardiographic data of 572 patients were analyzed and the results strongly indicated a direct relationship between MVO and NSVT in patients with HOCM.

NSVT is a common arrhythmia in HCM patients, with a prevalence rate of up to 20%–30% during a long‐term follow‐up period.^20^ In our study, the prevalence rate of NSVT was approximately 12.9% because only the latest Holter electrocardiogram recording before surgical treatment was analyzed among all patients. The frequency of NSVT is similar to that reported in Adaya's study, in which 77 patients underwent a 14‐day Holter electrocardiogram, and the prevalence rate of NSVT during the first 24 h was 16.9% despite the definition of NSVT being ≥3 beats at ≥120 beats/min.^21^ In addition, old age, maximal LV wall thickness, diabetes, and left atrial diameter were previously known to be associated with NSVT. Therefore, we focused on the effect of MVO on the development of NSVT in this study.

The mechanisms responsible for the development of NSVT in patients with MVO are not well understood. Several causes may increase the incidence of NSVT in HOCM patients with MVO. First, ischemia and myocardial fibrosis have been universally acknowledged to be associated with NSVT in patients with HCM. Patients with MVO may bring about high pressure in the apical cavity during the systolic and diastolic phases.^22^ The increased afterload on apical myocardium will increase its oxygen consumption and impair coronary flow through extravascular compression of the coronary artery, which leads to chronic myocardial ischemia.^22^ One study suggested that patients with severe cavity obliteration had lower myocardial flow perfusion than those with no or mild cavity obliteration during an exercise stress test.^22^ Long‐term myocardial ischemia is the main cause of myocardial fibrosis. In the presents study, 296 patients underwent cardiac magnetic resonance, and patients with MVO had a higher percentage of LV LGE than those without MVO. Cai also found a higher positive rate of myocardial fibrosis in HCM patients with MVO than those with apical HCM.^19^ In addition, the presence of MVO is associated with a higher possibility of the development of apical aneurysm, which has been demonstrated as a strong predictor of NSVT in HCM patients.^3^, ^4^, ^9^, ^18^ Studies have shown a prevalence of apical aneurysm of about 20% in patients with MVO during a long‐term follow‐up period.^3^, ^19^Although the mechanisms of the development of apical aneurysm in patients with MVO is not clearly understood, studies have indicated a relationship between apical aneurysm and a higher mid‐ventricular gradient.^9^, ^22^ In our study, four patients with MVO had an aneurysm on initial evaluation, with a prevalence rate of about 5.3%, and 2 of 4 patients had NSVT (one patients with a peak MVO gradient of about 58 mmHg, and the other 75 mmHg), which suggested that MVO patients with aneurysm may be prone to NSVT. These results may also explain our finding that the prevalence rate of NSVT increases with the severity of MVO. Nevertheless, the basic mechanisms responsible for the development of NSVT in patients with MVO remain unresolved. Further studies are needed to focus on the mechanistic link between MVO and NSVT in patients with HOCM.

MVO is a rare type of HCM, accounting for approximately 10% of cases.^23^ Patients with MVO had a higher incidence of NSVT, which was a significant risk factor of lethal cardiovascular events and sudden cardiac death (SCD), independent of its frequency, duration, and number of episodes than in those with isolated LVOT obstruction.^24^ Moreover, patients with MVO are also more prone to apical aneurysms, with a prevalence rate of up to >20% during a long‐term follow up. In a single‐center study, a peak MVO gradient >70 mmHg was the only independent risk factor for the formation of apical aneurysms.^9^ Moreover, patients with MVO had a higher incidence of syncope than those without MVO in the present study. According to recent guideline of HCM, patients with MVO are at high risk for SCD, in which NSVT, syncope, and apical aneurysm are important risks factors for SCD.^12^ Hence, it is extremely important to evaluate the timing of intervention in patients with MVO before the formation of apical aneurysm and to prevent patients with MVO from SCD.

Previous studies have evaluated the effects of several approaches to relieve obstruction in patients with MVO. In a large retrospective cohort study from the Mayo clinic, 196 patients with MVO underwent myectomy, including 124 who underwent combined transaortic and transapical myectomy, 64 who underwent transapical myectomy, and six who underwent transaortic myectomy. The results showed that after septal myectomy, the peak instantaneous gradient decreased remarkably, and the estimated 1‐, 5‐, and 10‐year survival rate were 99%, 98%, and 90%, respectively.^23^ Further, Tang and his colleagues analyzed the results of 40 HCM patients with MVO who underwent transaortic extended myectomy. After a median follow‐up period of 19 months, the patients' symptoms and heart function improved significantly, and only two patients had residual MVO due to inadequate myocardial resection.^24^ More importantly, Ryozo Maeda observed 6.2%/year of appropriate implantation cardioverter defibrillator intervention in MVO patients during a follow‐up period of 6.5 ± 5.1 years, which was much higher than that reported in a recent meta‐analysis of HCM patients overall (3.3%/year).^25^, ^26^ Therefore, septal myectomy is an effective treatment for patients with MVO to relieve mid‐ventricular cavity obstruction and improve symptoms and heart function. However, further investigation is needed to determine whether patients with MVO need ICD implantation. We expect that early diagnosis and appropriately timed treatments will reduce the occurrence of NSVT and the formation of aneurysms in patients with MVO and will eventually improve patients' symptoms and long‐term prognosis.

## 5 LIMITATION

The limitations of the present study are as follows. First, our study had a single‐center design with a small sample size of MVO patients. Hence, our study findings should be prudently extrapolated to other centers, and further studies with a larger population should be conducted to confirm our results. Second, due to the retrospective nature of data collection, some factors such as myocardial flow perfusion and myocardial fibrosis extension could not be evaluated in this study. This limited our ability to test the relationship between MVO and myocardial ischemia and myocardial fibrosis, which may explain the relationship between MVO and NSVT more effectively. Third, all patients underwent a 24 h Holter electrocardiogram only once before surgical treatment. Longer Holter electrocardiogram monitoring would have been more useful providing valuable information.

## 6 CONCLUSION

In conclusion, the presence and severity of MVO was significantly associated with higher incidence of NSVT in HOCM patients even though the relationship between MVO and the frequency, duration, and rate of NSVT episodes could not be demonstrated. Further studies are needed to focus on the potential mechanism linking between MVO and NSVT in HOCM patients.

## CONFLICT OF INTEREST

The authors declare that they have no competing interest.

## AUTHOR CONTRIBUTION

Changrong Nie and Shuiyun Wang contributed to the design of the study. Changrong Nie, Changsheng Zhu, and Minghu Xiao contributed to the data collection and analysis, while all authors contributed to data interpretation. Changrong Nie drafted the manuscript and Qiulan Yang, Yanhai Meng, and Rong Wu contributed significantly to the preparation. All the authors critically revised the manuscript, and gave final approval and agreed to be accountable for all aspects of the work, to ensure its integrity and accuracy.

## Supporting information


**Supplemental Table 1** NSVT characteristic in patients with and without MVO
