# Supplementary material for: Mid‐ventricular obstruction is associated with non‐sustained ventricular tachycardia in patients with hypertrophic obstructive cardiomyopathy
Source: Clin Cardiol. 2021 Feb 24;44(4):555–62. doi: 10.1002/clc.23575 (PMC8027587; doi:10.1002/clc.23575)
Supplement: Supplementary file 1 — Supplemental Table 1 NSVT characteristic in patients with and without MVO [file CLC-44-555-s001.docx]

| Supplemental table 1 NSVT characteristic in patients with and without MVO | | | |
| --- | --- | --- | --- |
| Variables | NSVT with MVO (N=17) | NSVT without MVO (N=55) | P |
| Number of Runs in 24-hour | 3.5 ± 4.8 | 4.8 ± 13.4 | 0.686 |
| Number of beats longest run | 7.5 ± 4.7 | 7.0 ± 6.5 | 0.790 |
| Rate fastest run(beats/min) | 125.7 ± 15.3 | 134.2 ± 24.9 | 0.187 |

Values are expressed as mean ± SD. Abbreviations as in Table 1.
